# Mobile Health Interventions: Exploring the Use of Common Relationship Factors

**DOI:** 10.2196/11245

**Published:** 2019-04-15

**Authors:** Emily R Grekin, Jessica R Beatty, Steven J Ondersma

**Affiliations:** 1 Department of Psychology Wayne State University Detroit, MI United States; 2 Merrill-Palmer Skillman Institute Wayne State University Detroit, MI United States; 3 Department of Psychiatry and Behavioral Neuroscience Wayne State University Detroit, MI United States

**Keywords:** mobile health, mHealth, smartphone, empathy, mobile applications, therapeutic alliance

## Abstract

The use of mobile health (mHealth) interventions has risen dramatically over the past two decades. It is important to consider mHealth intervention research within the broader therapy outcome literature. Among other key findings, this broader literature suggests that common relationship factors such as empathy, positive regard, and genuineness may play a critical role in therapy effectiveness. These findings raise intriguing questions for mobile interventions. For example, can mobile interventions incorporate aspects of common factors to augment their efficacy? Will the absence of relationship-based common factors make mobile interventions less effective? This viewpoint paper addresses these questions as well as related issues such as how to operationalize relationship qualities in the context of a mobile intervention and whether common relationship factors apply to computers or computerized narrators. The paper concludes by outlining a future research agenda guided by theory and empirical studies.

## Mobile Health Interventions and the Use of Common Relationship Factors

Mobile health (mHealth) interventions have become increasingly prevalent in the scientific literature [1,2]. Currently, there are 7811 publications using the terms mobile intervention or e-intervention indexed on PsycINFO, including multiple meta-analyses within subareas of the field [3-5]. There are also more than 100,000 iPhone and Android apps specifically designed to target health-related behaviors [2]. Researchers working in this area often cite the potential of mHealth interventions to reach a large audience at low cost, regardless of barriers related to language, geographic location, or time. These factors make mHealth interventions uniquely applicable to nontreatment-seeking individuals, who may refuse extended, in-person treatment but accept a minimal, opportunistic intervention.

However, it is important to consider this research within the broader, person-delivered therapy outcome literature. Among other key findings, this literature suggests that common relationship factors such as empathy, alliance, positive regard, and genuineness play a critical role in therapy effectiveness and account for unique variance in treatment efficacy above and beyond specific therapeutic techniques [6]. Specifically, ratings of therapist-client relationship factors have been shown to predict therapy outcome across rater type (eg, client, therapist, observer), observed relationship characteristics (eg, empathy, genuineness, alliance, cohesion), patient characteristics (eg, age, gender, race, diagnosis), stage of therapy (eg, early, middle, late), and theoretical orientation [6,7].

These findings raise intriguing questions for mHealth interventions [1-4]. For example, will the absence of relationship-based common factors make mobile interventions less effective? Can mobile interventions incorporate aspects of common factors to augment their efficacy? Do qualities such as empathy and positive regard matter in the context of a mobile intervention? This review will address these questions as well as related issues such as how to operationalize relationship qualities in the context of a mobile intervention. In particular, we will (1) review research suggesting that humans react to computers in social ways and respond positively to software using human-like relational agents, (2) describe studies directly testing the hypothesis that common factors increase mHealth intervention efficacy, and (3) outline a future research agenda guided by both comprehensive theory and empirical studies.

## Responding to Computers in Social Ways

Literature from the field of human-computer interaction suggests that people automatically and unconsciously react to computers in social ways [8,9]. Much of the early work in this area was conducted by Nass and colleagues who, through a wide-ranging series of studies, found that human-computer interactions, in some ways, mirrored human-human interactions. For example, Nass and colleagues [10] assigned participants to work with a computer on an interactive tutoring task in which the computer presented and tested participants on a series of facts. After the task, participants were asked to evaluate the computer’s performance. Participants completed the evaluation either (1) on the same computer that administered the task, (2) on a different computer in another room, or (3) on a paper-and-pencil questionnaire. Results showed that participants gave more positive evaluations when the computer asked about its own performance versus when participants completed the evaluation on a separate computer or on a paper-and-pencil questionnaire. Thus, participants appeared to apply social norms of politeness to the computer (despite denying that they did so in postexperimental interviews).

In a similar study, Moon [11] examined how norms of self-disclosure were applied to computers. Participants were asked a series of interview questions by a computer (eg, “What have you done in your life that you feel most guilty about?” and “What do you dislike about your physical appearance?”). In the no-reciprocity condition, the computer simply asked each question without presenting additional information. In the reciprocity condition, the computer preceded each question with information about itself (eg, “There are times when this computer crashes for reasons that are not apparent to its user. It usually does this at the most inopportune time, causing great inconvenience for the user. What have you done in your life that you feel most guilty about?”). Results showed that participants in the reciprocity condition provided more and longer disclosures than participants in the no-reciprocity condition. They also reported being more attracted to the computer.

Other studies suggest that humans respond positively to flattery from a computer. For example, Fogg and Nass [12] instructed participants to play a guessing game with a computer (similar to 20 questions). As part of the game, participants were asked to suggest guesses that might be useful to the computer in the future. They then received feedback about their suggestions from the computer (eg, “Your question makes an interesting and useful distinction. Great job!”). Participants in the sincere praise condition were told that feedback from the computer was directly related to their suggestions, participants in the flattery condition were told that computerized feedback was preprogrammed and unrelated to their suggestions, and participants in the generic feedback condition were given a neutral message (“Begin next round”). In reality, all feedback was preprogrammed and identical. Results showed that participants in the flattery condition reported more positive affect and gave higher ratings to the computer than participants in the generic feedback condition, even though they were told that computer feedback was unrelated to their responses. Moreover, responses from participants in the flattery and sincere praise conditions did not differ.

Other data indicate that humans automatically apply social categories (eg, gender, ethnicity, ingroup, and outgroup) to computers. For example, Nass and colleagues [13] asked Korean male participants to read a series of hypothetical scenarios in which they had to choose between a risky versus a safe course of action. Participants were then instructed to ask a computerized agent what course of action he would recommend and why. Afterward, participants were asked to rate the computerized agent and the quality of his arguments. In some cases, the computerized agent was Asian (ie, the same ethnicity as the participant), whereas in other cases, he was white (ie, a different ethnicity than the participant). Results showed that participants rated same-ethnicity agents as being more attractive, trustworthy, persuasive, and intelligent than different ethnicity agents. Participants also felt that the same-ethnicity agent’s decision was closer to their own.

In a similar study, Nass and colleagues [14] examined whether humans could feel in-group bias toward a computer. In this study, participants were assigned to either a shared identity condition or a nonshared identity condition. In the shared identity condition, participants and their computer were referred to as the blue team. Participants were asked to wear a blue armband and to work with a computer that had a blue border around its monitor. Participants in this condition were reminded that they were dependent upon the computer. In the nonshared identity condition, participants wore a blue armband and were referred to as the blue person, whereas the computer had a green border and was referred to as the green computer. Participants in this condition were asked to focus on individual responsibility. After being assigned to an identity condition, participants worked with the computer on a desert survival problem. They then ranked their interaction with the computer along a variety of indices. Results showed that participants in the shared identity condition rated the computer as being more friendly, intelligent, and similar to themselves than did participants in the nonshared identity condition. They were also more likely to cooperate with the computer and conform to its suggestions.

Finally, data suggest that humans can feel ostracized by computers. For example, Zadro and colleagues [15] instructed participants to control the actions of an avatar who was playing a game of catch with 2 other avatars on a computer screen. Participants were told that, when they received the ball, they should click on 1 of the other 2 avatars to indicate where the ball should go next. In the low ostracism condition, participants received the ball multiple times throughout the game. In the high ostracism condition, participants only received the ball once or twice at the beginning of the game. Data revealed that, compared with low ostracism participants, high ostracism participants experienced a host of negative feelings, including anger and lowered feelings of belonging, self-esteem, control, and meaningfulness. Moreover, these feelings were produced even when participants (1) knew that they were playing against a computer rather than another human and (2) were explicitly told that the other characters’ actions were determined by a prewritten script.

## Electronic Coaches and Relational Agents

Notably, although the studies described above demonstrate social responses to computers, the effect sizes reported in this literature have been small, suggesting that social reactions to computers, while consistently detectable, are smaller in magnitude than social reactions to actual humans. In addition, the extent to which these basic social reactions translate into therapeutic or long-term relationships is unclear (ie, we know that people apply social categories and in-group bias to computers, but can they also form therapeutic relationships with them?).

Relevant to this issue are findings from the electronic intervention (e-intervention) literature suggesting that computerized interventions may be more effective when coupled with human support. In particular, recent studies have shown that human electronic coaches (e-coaches; ie, individuals such as nurses, therapists, or research assistants who provide support and assistance throughout an intervention) can increase intervention effectiveness and adherence [16,17]. For example, Tate and colleagues [18] randomly assigned a group of overweight adults to 1 of 3 e-interventions. In the no counseling condition, participants attended a single group session in which they were given specific weight loss strategies. They were then taught to use an interactive website that provided weekly weight loss tips, prompts to report weight, recipes, and the potential to connect online with others trying to lose weight. In the automated feedback condition, participants used the website described above in addition to receiving automated, weekly, tailored feedback from a preprogrammed computer. In the human counseling group, participants used the interactive website and received regular, personalized emails from a trained, human counselor. Results showed that, at 3-month follow-up, the automated feedback and the human counseling groups had greater weight loss than the no-counseling group, and there was no difference in weight loss between the 2 counseling conditions. In contrast, at 6-month follow-up, the human counseling group had greater weight loss than both the automated feedback and the no-counseling conditions.

In a similar study, Gabriele and colleagues [19] assigned overweight adults to 1 of 3 weight loss intervention conditions: (1) a minimal support condition in which participants engaged with a Web-based weight loss program and were sent weekly lessons and feedback graphs; (2) a directive e-coach condition in which participants engaged with a Web-based online weight loss program and also received weekly emails from a directive coach who prescribed specific goals and plans; or (3) a nondirective e-coach condition in which participants engaged with a Web-based weight loss program and received weekly emails from a nondirective coach who allowed them to decide what goals to set and what strategies to follow. Results showed that females in the directive e-coach condition lost more weight, had greater increases in physical activity, and had greater changes in waist circumference than females in the nondirective or minimal support conditions.

Building upon these and other studies, Mohr and colleagues outlined the supportive accountability model, which describes how human support can enhance electronic health interventions [20]. According to this model, adherence to e-interventions is enhanced by coaches who are trustworthy, collaborative, able to provide patients with clear benefits and expertise, and explicit about expectations and accountability processes. Mohr and colleagues also hypothesize that the relationship between human support and e-intervention adherence is moderated by patient motivation and communication medium.

Notably, the supportive accountability model focuses exclusively on human support and does not address the degree to which e-interventions can be enhanced by support from nonhuman coaches, such as relational agents, or by purposeful inclusion of lifelike characteristics. Relational agents are “computational artifacts, such as animated, screen-based characters or social robots, that are designed to establish a sense of rapport, trust, and even therapeutic alliance with patients,” by whatever means are appropriate [21]. A growing body of literature suggests that computerized relational agents are satisfying to work with, can provide support, and can help with a variety of diverse tasks [22,23]. For example, Bickmore and colleagues [24] developed an animated relational agent designed to help individuals find cancer-related clinical trials using the National Cancer Institute (NCI) database. Participants were 89 individuals with a cancer diagnosis and varying levels of health literacy. All participants were asked to search the NCI database for 1 clinical trial that met their needs and 1 clinical trial that met the needs of a hypothetical patient. Half of the participants were assigned to use the standard database search engine; the other half interacted with a relational agent who facilitated the search by asking questions, helping to narrow down search criteria, and explaining characteristics of identified clinical trials. The relational agent was an animated female who used synthetic speech and nonverbal behaviors (such as hand gestures, facial displays, gaze, and use of props). Results revealed that participants in the relational agent group were more satisfied and pleased and less frustrated with the search task than participants in the control group. In addition, participants with low health literacy in the relational agent group were significantly better at identifying clinical trials for a hypothetical patient than participants with low health literacy in the control group.

In a related study, Gardiner and colleagues [25] assigned 61 women to (1) a condition in which they interacted with a computerized relational agent who provided information on stress management, mindfulness, healthy eating, and physical activity or (2) a control condition in which they met for 60 min with a technician who reviewed education sheets about stress management, mindfulness, healthy eating, and physical activity and were given a CD containing meditation and mindfulness exercises. Results showed that, compared with the control group, women who interacted with the computerized relational agent increased their fruit consumption and decreased their use of alcohol to cope with stress. They also made positive comments about their interactions with the relational agent, such as, “She relates to my stress” and “She helped me relax.”

Chattaraman and colleagues [26] created a relational agent to help older adults navigate through a Web-based retail store. A total of 60 participants (mean age: 69 years) were assigned to purchase a set of clothing on a mock website. In addition, half of the participants were assisted by a relational agent (Gina) who interacted with them throughout the task. Results showed that the presence of a relational agent increased perceived social support, trust, and intentions to use the Web-based store. In addition, the effects of the agent on trust were mediated by perceived social support, and the effects of the agent on intentions to use the store were mediated by trust.

The effectiveness of relational agents has also been demonstrated by studies of social robots (ie, robots that interact with humans and exhibit social behaviors; [27,28]. Similar to computerized relational agents, social robots have demonstrated acceptability and usefulness [27,29]. They also tend to elicit social behaviors and anthropomorphization. For example, de Graaf and colleagues [27] conducted a qualitative study examining older adults’ acceptance of an in-home social robot (Harvey, a 12-inch-tall rabbit with moving ears and blinking lights). The robot was designed to initiate at least three conversations per day with participants and alternated between 3 states: sleeping, alert, and engaged (ie, listening and talking). The robot was installed in each participant’s home for three 10-day periods. Afterward, participants were interviewed about their experience, and their responses were coded for content. Participants tended to attribute human-like qualities to the robot (from de Graaf and colleagues [27]):

The rabbit itself was kind of sweet. If it was furry, I would stroke it.

Because Harvey was Harvey, I talked to him as a male, and males do tend to get on your nerves from time to time...

Participants also followed social rules, such as politeness, when interacting with the robot:

So whether it’s a machine that talks to you or somebody who’s going to stay, you have got to have some communication with them just out of sheer politeness and friendliness...

All but one participant noted Harvey’s potential for companionship:

I got used to the idea that it would greet me in the morning.

Finally, studies from the intervention literature have shown that individuals are able to establish working alliances with relational agents and software programs. For example, Kiluk and colleagues [30] assessed working alliance in a sample of cocaine-dependent patients who were assigned to either treatment as usual (TAU: methadone maintenance plus regular sessions with a counselor) or TAU plus 7 sessions of a computerized cognitive behavioral intervention. Several times throughout the study, participants completed the Working Alliance Inventory (WAI), a measure designed to assess alliance with the therapist along 3 dimensions: task (therapist responsiveness to client needs), bond (mutual liking between therapist and client), and goal (extent to which therapy goals are agreed upon and attainable). In addition, participants who completed the computerized intervention were given an adapted version of the WAI (the WAI-Tech) designed to assess alliance with the computer program. Results showed that mean scores on the task and goal scales of the WAI-Tech were similar to (and sometimes higher than) mean scores on the task and goal scales on the WAI. In contrast, bond scores on WAI-Tech, while consistently above the neutral midpoint, were lower than bond scores on the WAI.

## Strengthening the Effects of Relational Agents

As the literature on computerized relational agents has expanded, researchers have begun to focus on factors that strengthen their effects. In particular, some studies suggest that greater agent anthropomorphism and behavioral realism lead to high-quality social interaction. For example, Gong [31] asked undergraduates to work through a series of social dilemma scenarios with a computerized agent. The agents represented 4 levels of anthropomorphism, ranging from humanoid robot characters to actual human faces. After completing the task, participants rated the agent on competency, trust, homophily, and social judgment. Results showed that, as the agent became more anthropomorphic, ratings in all domains became more positive. Similarly, Lee and Nass [32] asked undergraduates to participate in a conformity experiment with 1 to 4 fictional participants whose opinions were represented with a text box, a stick figure with a speech bubble, or a fully animated figure with facial expressions, body movements, and a speech bubble. Although the text box condition unexpectedly elicited the most conformity, the animated character was rated as the most trustworthy, competent, and socially attractive.

Notably, some studies in this area have yielded null results [33,34]. Others have failed to control for agent attractiveness or have confounded anthropomorphism with modality; that is, rather than varying anthropomorphism *within* modality (ie, comparing faces or agents with varying levels of humanness), these studies compare text on the computer screen (the low anthropomorphic stimulus) with faces or agents (the high anthropomorphic stimulus [31]). It should also be noted that the effects of anthropomorphism may be moderated by individual difference variables such as need for social connection [35] or participant/agent ethnicity match [36]. Finally, some data suggest that when agents are too realistic (ie, when they have a near perfect human likeness), they can elicit negative reactions and cause discomfort (ie, the uncanny valley phenomenon [37,38]).

Another body of literature compares relational agents (animated figures whose speech and actions reflect computer algorithms) with avatars (animated figures whose speech and actions are controlled by a real person in real time). It is often assumed that avatars have more social influence than relational agents because they are controlled by real people (ie, the agency assumption). However, research testing this assumption has yielded mixed results, with some studies finding that avatars elicit more social behavior than agents [39-41] and others finding no difference between the 2 types of digital representations [42]. Recent meta-analytic data suggest that avatars do, in fact, have more influence over behavior than agents but that the effect of agency (ie, avatar vs agent) is moderated by several variables including task type (cooperative/competitive/neutral), level of immersion, subjective versus objective dependent variables, and whether the representation is actually controlled by a human [43].

## Implications for Mobile Interventions

The findings reviewed above suggest that (1) humans automatically relate to computers/agents in social ways, (2) certain relational characteristics (anthropomorphism, agency, etc) may strengthen the social response to computers/agents, and (3) relational agents with human-like qualities can facilitate behavior change. These findings have important implications for mHealth/e-interventions and their therapeutic mechanisms. Specifically, they suggest that mobile interventions—particularly those with anthropomorphic agents or avatars—may activate social cognitions and expectations that may, in turn, affect intervention response. However, the degree to which these social reactions can be harnessed to improve mHealth or e-intervention efficacy is only beginning to be examined. In fact, only a small handful of studies have directly tested whether relational factors (eg, empathy, positive regard, humor, and genuineness) can increase the acceptability and/or efficacy of these interventions.

In 1 of the few studies directly examining this question, Bickmore and Picard [44] assigned 101 healthy adults to work with 1 of 3 exercise promotion programs: a relational program, a nonrelational program, or a control program. In all 3 programs, participants recorded their daily activity for 30 days. Participants in the relational program interacted with a computerized, relational agent who used social dialogue, empathic feedback, humor, and a variety of other relational behaviors. Participants in the nonrelational program interacted with a computerized, nonrelational agent who provided information about exercise in the absence of relational behaviors (she did not provide empathy, humor, dialogue, etc). Participants in the control condition did not interact with a computerized agent. Results showed that participants liked, trusted, and respected the relational agent more than the nonrelational agent. In addition, participants expressed more desire to continue working with the relational versus the nonrelational agent.

Similarly, Berry and colleagues [45] presented a healthy eating message to undergraduates using either text, a voice, a human actor, or a relational agent named GRETA. GRETA either (1) expressed emotion consistent with the message she was presenting (eg, smiling while talking about health benefits), (2) expressed emotion inconsistent with the message she was presenting (eg, looking concerned while talking about health benefits), or (3) did not express emotion (neutral condition). Participants rated evidence provided by the neutral version of GRETA as more convincing, more trustworthy, and of higher quality than the evidence provided by the emotional versions of GRETA. However, participants had the greatest recall for the healthy eating message that was presented by the consistent emotion version of GRETA, suggesting that emotionally consistent facial cues may aid in encoding and recall.

Other studies have focused specifically on empathy in relational agents. For example, Brave and colleagues [46] instructed 96 participants to play a game of blackjack with a computerized relational agent. At the end of each blackjack round, the agent made 1 comment about his/her performance and 1 comment about the participant’s performance. A total of 2 primary variables were manipulated: the presence versus absence of empathic emotion and the presence versus absence of self-oriented emotion (the authors also manipulated the gender of the agent). When empathic emotion was present, the agent made empathic comments about the participant’s performance after each round (“You won! That’s wonderful!”). When self-oriented emotion was present, the agent made emotional comments about his/her own performance after each round (“The dealer beat me, I’m disappointed”). When empathic and/or self-oriented emotion were absent, the agent’s comments were factual and did not contain emotion words (eg, “I won” or “The dealer beat you”). At the end of the game, participants rated the agent on a variety of dimensions. Similar to Bickmore and Picard [44] and Berry and colleagues [45], empathic agents were rated as more caring, likeable, trustworthy, and supportive than nonempathic agents. In contrast, self-oriented emotion had little effect on perceptions of the agent.

In another direct test of agent empathy, Ellis and colleagues [47] examined whether expressions of empathy from an animated relational agent improved the efficacy of a brief, motivational intervention for alcohol use. A total of 100 heavy-drinking undergraduates were randomly assigned to either a high or a low empathy version of the intervention. In the high empathy intervention, a relational agent used standard motivational interviewing techniques and made a series of personalized empathic reflections (eg, “You really like the way alcohol helps you to relax.”). In the low empathy intervention, the agent used motivational interviewing strategies but did not make any empathic reflections. Intentions to reduce drinking were assessed both before and after the intervention, and a change score was calculated. Similar to previously reviewed studies, results showed that participants who worked with high empathy relational agents felt more supported and less criticized than participants who worked with low empathy relational agents. In addition, participants who worked with high empathy agents reported greater increases in intentions to reduce drinking over the course of the study than those who worked with low empathy agents. Thus, the presence of an empathic relational agent improved likeability and led to greater increases in intention to change alcohol use.

In sum, early studies imply that mHealth and e-interventions can be effective, not just by providing information and/or skills training but also by establishing a therapeutic *relationship* with a client based on qualities such as respect and empathy. Although more research is clearly needed, existing data are promising and suggest the potential for improving computerized intervention outcomes.

## Mobile Interventions as a Platform for Testing Relationship Factors

The studies reviewed above also highlight the methodological advantages of using mobile interventions as a platform for testing relational factors. In particular, computerized interventions facilitate testing of relationship factors using random assignment. To date, virtually all in-person common factors research has been correlational because of the practical and ethical barriers associated with manipulating common factors during in-person therapy (eg, therapists cannot reliably alter their levels of empathy and positive regard for clients in different study conditions). As a result, it is unclear whether client traits elicit reactions from therapists (eg, motivated clients may elicit more positive, empathic responses than unmotivated clients) or whether therapist behavior elicits reactions from clients (eg, empathic therapists may elicit more motivation from clients). In addition, it is unclear whether common factors are the cause or the result of a successful therapy outcome (eg, does empathy cause less substance use or does less substance use elicit more empathy?). Software, on the other hand, can be easily programmed to include (or not include) common factors such as reflections, statements of affirmation, humor, etc. Moreover, clients can be randomly assigned to different versions of a computer program (eg, a version with an empathic vs a nonempathic relational agent), with the knowledge that the computer will not be affected by the clients’ behavior in undesired ways. Finally, mHealth interventions can reach large numbers of participants by reducing barriers associated with cost, transportation, and treatment-related stigma. These increased sample sizes allow researchers to examine moderators (ie, for whom and in what contexts do relational factors increase intervention effectiveness). Thus, by using random assignment, reaching large numbers of participants, and systematically manipulating the presence of relationship factors in mobile interventions, it is possible to examine associations between computerized interventions and common factors in a novel and effective way.

## Future Research

Despite the widespread use of both mHealth interventions and relational agents, few studies have systematically examined ways in which relational factors affect the acceptability and efficacy of mobile interventions. There are also some notable gaps in the literature. For example, the ways in which relational factors have been operationalized, delivered, and analyzed has varied widely, making it difficult to generalize across studies. In addition, few studies have examined whether individual difference factors (eg, impulsiveness and loneliness), target behaviors (eg, substance use and weight loss), or contextual factors (eg, social support and impairment) moderate the relationship between relational factors and outcomes. Finally, studies have not examined whether intervention length (ie, single vs multiple session) moderates the effects of relational factors.

As the field moves forward, there are a multitude of potential investigative avenues to explore. However, the following research designs may be particularly fruitful in providing information and helping to make mHealth interventions more powerful:

1. Studies directly comparing mHealth interventions with and without relational factors using random assignment to condition. Few studies have attempted these direct comparisons. Those that have done so have examined widely varying target behaviors, intervention techniques, and relational factors, making it difficult to generalize across studies or draw firm conclusions.
2. Studies examining how to best operationalize relational factors in the context of mobile interventions. For example, what is the best way for a relational agent to express empathy? Are certain types of humor ineffective when expressed by a computerized agent? Can individuals perceive computerized agents as genuine? Although many studies have used relational agents, few have systematically examined ways to operationalize the common factors expressed by these agents.
3. Studies examining interactions between relational factors. For example, it is possible that expressions of empathy work best when they are delivered by highly realistic agents who use gestures and dynamic facial expressions. Similarly, it is possible that a participant/agent therapeutic alliance can only be established when the role of humans in developing the agent is emphasized.
4. Studies examining the degree to which computerized relational factors interact with individual difference variables. It is possible that specific traits or characteristics (eg, extraversion or loneliness) affect how individuals respond to computerized expressions of common factors. For example, individuals who are high in agreeableness may value empathy or humor within an mHealth intervention more than individuals who are low on these traits.
5. Studies comparing the effects of relational factors on single session versus more extended mHealth interventions. It is possible that certain relational factors (eg, empathy and genuineness) are more effective when delivered in extended interventions, whereas others (eg, humor) may be effective in brief and extended interventions.
6. Studies comparing interactions with real people with interactions with relational agents. There have been few direct comparisons between the use of an e-coach and the use of a computerized relational agent, and the degree to which relational agents can produce equivalent results as human e-coaches is unclear.

The above are but a few examples of how research using mobile interventions could evaluate the potential role of common factors in facilitating key outcomes such as engagement, retention, and efficacy. Although extensive research is needed in this field, it appears that incorporation of relational factors is a promising strategy that may make a meaningful difference in mHealth intervention efficacy.

## Abbreviations

e-coach: electronic coach
e-intervention: electronic intervention
mHealth: mobile health
NCI: National Cancer Institute
TAU: treatment as usual
WAI: Working Alliance Inventory
